# Associations between toe grip strength and hallux valgus, toe curl ability, and foot arch height in Japanese adults aged 20 to 79 years: a cross-sectional study

**DOI:** 10.1186/s13047-015-0076-7

**Published:** 2015-05-02

**Authors:** Daisuke Uritani, Takahiko Fukumoto, Daisuke Matsumoto, Masayuki Shima

**Affiliations:** Department of Physical Therapy, Faculty of Health Science, Kio University, 4-2-2 Umaminaka, Koryocho, Kitakatsuragigun, Nara 6350832 Japan; Department of Public Health, Hyogo College of Medicine, 1-1 Mukogawacho, Nishinomiya, Hyogo 6638501 Japan

**Keywords:** Toes, Foot, Muscle strength, Hallux valgus, Range of motion, Sex, Aging

## Abstract

**Background:**

The associations between toe grip strength (TGS) and foot structure are not well known, although foot structure is inferred to affect TGS. This study investigated the associations between TGS and hallux valgus angle (HVA), toe curl ability, and foot arch height (FAH).

**Methods:**

This study analysed 227, 20 to 79-year-old, community-dwelling participants. TGS, HVA formed by the first metatarsal bone and the proximal phalanx of the hallux, toe curl ability (percentage) calculated as (foot length–flexed foot length)/foot length, and FAH (percentage) calculated as navicular height/truncated foot length were measured. To elucidate associations between TGS and foot structure, a correlation analysis and stepwise multivariate linear regression analyses were performed, based on the participant’s sex. Pearson’s correlation coefficients for TGS with age, height, weight, HVA, toe curl ability, and FAH were also calculated. In the stepwise, multivariate linear regression analyses, the independent variable was TGS and the dependent variables were those that significantly correlated with TGS, as shown by the Pearson’s correlation coefficients. The significance level was set at 5%.

**Results:**

According to the Pearson’s correlation coefficients, in men, TGS was significantly correlated with age, height, toe curl ability, and FAH. According to the stepwise multiple regression analysis, TGS correlated with age and toe curl ability (adjusted R^2^=0.22). In women, TGS was significantly correlated with age, height, and toe curl ability (adjusted R^2^=0.40).

**Conclusions:**

TGS was associated with toe curl ability in both men and women. However, TGS was not associated with HVA and FAH in men or women. The results of this study may lead to the development of effective interventions to improve TGS. However, factors other than structure of the foot require more detailed investigation to clarify the factors contributing to TGS.

## Background

The performance characteristics of the toe, such as toe flexor strength, are very important for various movements, including standing [1] and walking [2,3]. Menz et al. [4] demonstrated that toe flexor strength was a significant independent predictor of balance and functional ability in older people. They also reported that people who experienced falls exhibited decreased toe flexor strength compared with those who did not fall [5]. However, toe flexor strength has not been evaluated in a standard manner similar to that used to determine hand grip strength.

We previously assessed age-related changes in toe grip strength (TGS) and investigated relationships between TGS and sex, age, weight, and height using a toe grip dynamometer [6]. We found that these factors contribute to the prediction of TGS; however, the association of other factors, such as foot structure characteristics, with TGS are unknown.

Structurally, toe deformities, such as hallux valgus, are often found clinically, regardless of patient age or sex. Mickle et al. [7] demonstrated an association between toe deformity and reduced toe flexor strength. A relationship between the toe flexor strength and the medial longitudinal arch (MLA) height has also been reported [8,9]. Weak plantar intrinsic or extrinsic muscles (i.e., toe flexors) that do not provide sufficient dynamic truss support for the MLA may be involved in the underlying aetiology of decreasing foot arch height (FAH) [8]. Headlee et al. [10] reported that repeated isotonic flexion of the metatarsophalangeal joints, through their full range of motion, resulted in navicular drop. A loss of foot intrinsic muscular function due to fatigue has also been reported to result in a loss of structural MLA support [11]. However, some studies have reported that there is no association between TGS and the MLA height [12,13]. Therefore, the association between TGS and FAH remains unclear. Additionally, although some researchers have reported that the range of toe flexion (a factor in toe curl motion) is related to TGS in younger women [9] and in frail, elderly women [13], the association between TGS and range of toe flexion remains to be clarified in other groups.

Factors including hallux valgus, toe curl ability, and FAH might also influence TGS; however, the associations between TGS and these characteristics are not well known. These characteristics can be easily measured in clinical practice, and might be amenable to interventions that improve these conditions and lead to improved TGS. Therefore, the present study investigated the association between TGS and foot characteristics, including hallux valgus, toe curl ability, and FAH. We hypothesised that reduced severity of hallux valgus, greater toe curl ability, and FAH were associated with greater TGS.

## Methods

### Participants

Participants were community-dwelling volunteers, 20 to 79-years-old, recruited at municipal events conducted for the measurement of physical fitness; the events were managed by Kashihara City and Koryo Town, in Nara, Japan. Data were collected between September 2011 and September 2012. None of the participants had known neuromuscular or musculoskeletal pathologies, or used walking aids. Prior to the study protocol measurements, the histories of the participants were obtained; based on their histories, the need for walking devices was ruled out. Toe deformities were confirmed by inspection; severe hallux valgus was defined as grade 4 according to the Garrow et al. grading system [14]. Individuals with severe hallux valgus and those who could not perform TGS measurements were not analysed.

The research ethics committee of Kio University approved the study (H23-8), and each participant provided written informed consent prior to participating in the study.

### Experimental protocol

A toe grip dynamometer (T.K.K.3362; Takei Scientific Instruments, Niigata, Japan) was used to measure TGS; the reliability of the instrument has been previously reported [15]. According to our study, the substantial intra- and inter-rater reliabilities of the toe grip dynamometer, based on the criteria by Landis and Koch, indicate that it is suitable for clinical use [16]. The usefulness of the equipment in clinical settings and field research, for people 20 to 79-years-old, has been proven [15]. The measurements performed in this study were similar to those reported in our previous study [6,15]. Participants practiced the testing procedures, using submaximal effort, before the actual measurements were taken. We measured TGS on the supporting lower extremities because we speculated that TGS may be more important for supporting the body during activities that require standing on one leg, such as walking and standing while shifting one’s weight to the preferred side. The supporting lower extremity was identified as being contralateral to the one preferred for kicking a ball. TGS was measured twice, at approximately 10-second intervals, and the mean value was calculated. The measurements were performed while the participants were rested and not fatigued.

**Figure 1:**
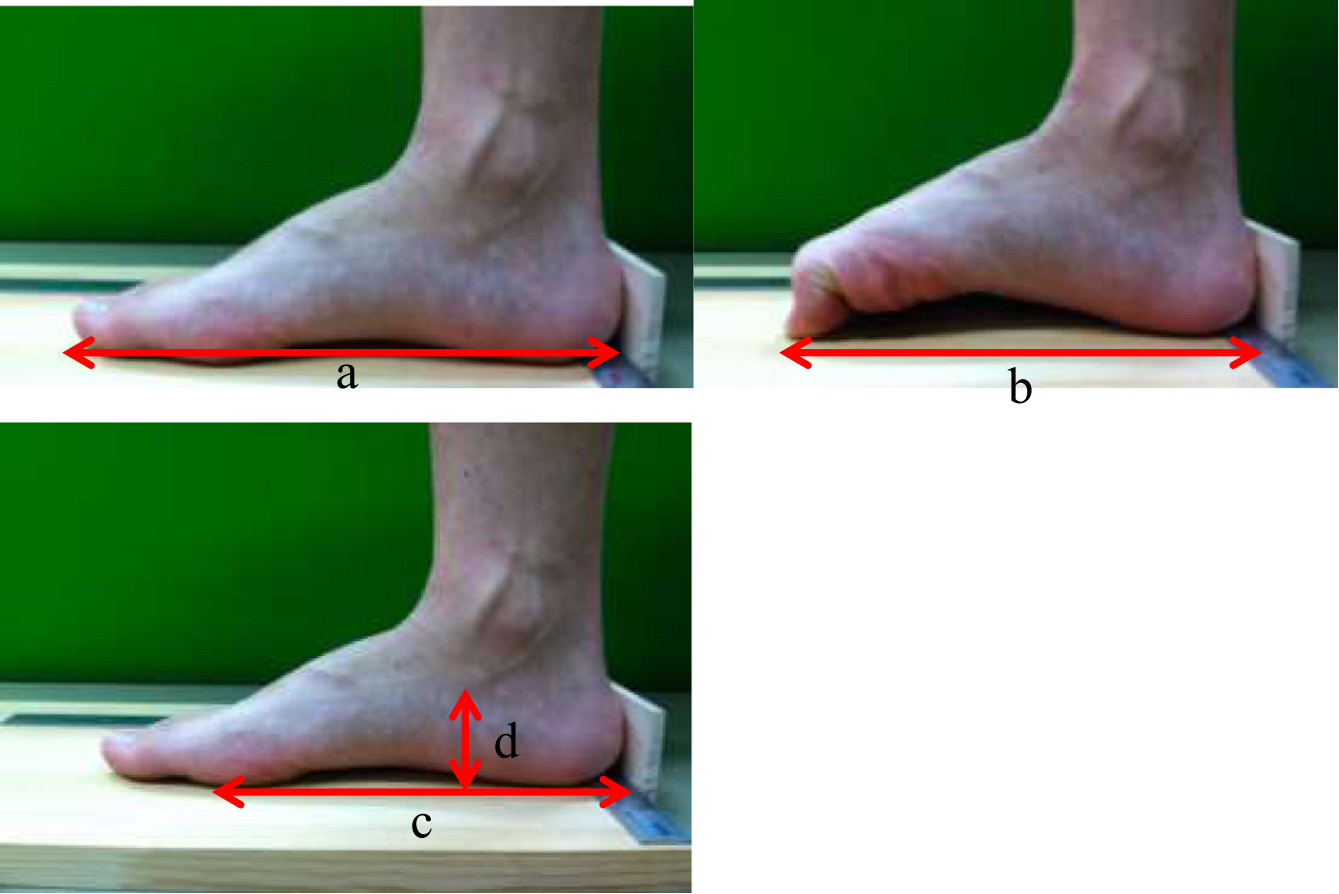
Foot characteristic measurements. **a**, foot length; **b**, flexed foot length was measured as the length from the heel to the tip of the hallux with maximal toe flexion; **c**, truncated foot length was measured as the length between the first metatarsal head and heel; **d**, navicular height was measured as the length from the floor to the top of the navicular tuberosity.

The hallux valgus angle (HVA) was defined as the angle formed by the first metatarsal bone and the proximal phalanx of the hallux; it was measured using a goniometer. The foot length, flexed foot length (distance from the heel to the tip of the hallux with maximal toe flexion), truncated foot length (distance between the first metatarsal head and the heel), and navicular height were measured using a scale (Figure 1), with the participant in a sitting position. Toe curl ability (percentage) was calculated as (foot length–flexed foot length)/foot length, using a modified method for measuring toe curl ability [9]. Toe curl ability consists of the range of toe flexion motion, involving the hallux, and arch contraction. The reliability of toe curl ability measurements was calculated for a separate group of 15 participants in the same age range; we noted that the intrarater reliability (ICC_1.1_) was 0.79 and the interrater reliability (ICC_2.1_) was 0.95. Greater values for toe curl ability indicate greater toe and foot mobility, i.e., greater toe and foot flexion. FAH (percentage) was calculated as the navicular height/truncated foot length [9]. Greater FAH values indicate greater MLA heights.

### Statistical analysis

In the present study, 227 individuals, with hallux valgus grades 1 to 3, (49 men and 178 women; mean age [standard deviation], 61.0 [14.2] years) [14] were analysed.

Unpaired *t*-tests were performed to compare outcome measures, HVA, toe curl ability, and FAH in men and women. To elucidate the associations between TGS and other variables, correlation analyses and stepwise multivariate linear regression analyses were performed, according to participant sex, to exclude any gender bias. Pearson’s correlation coefficients for TGS with age, height, weight, HVA, toe curl ability, and FAH were calculated. In the stepwise multivariate linear regression analyses, the independent variable was TGS and the dependent variables were those that were significantly correlated with TGS, according to the Pearson’s correlation coefficient. We also determined the adjusted R^2^ value for the multivariate linear regression analyses. The significance level was set at 5% for all determinations, and all data were analysed using IBM SPSS statistics 22.0 (IBM Japan, Tokyo, Japan).

## Results

Participant characteristics are shown in Table 1. A large proportion of the participants were over 60 years of age. Although significant differences were found between men and women with respect to foot length, flexed foot length, truncated foot length, and navicular height, there were no significant differences in HVAs, toe curl abilities, and FAHs between the sexes (Table 2).

**Table 1:** **Participant characteristics**

	**Total**	**Men**	**Women**
	(N=227)	(N=49)	(N=178)
Age group (years)			
20 to 29	16	6	10
30 to 39	9	4	5
40 to 49	11	3	8
50 to 59	19	5	14
60 to 69	107	17	90
70 to 79	65	14	51
Age (years)	61.0 (14.2)	56.7 (17.1)	62.2 (13.0)
Height (cm)	156.1 (8.4)	167.2 (6.0)	153.1 (6.2)
Weight (kg)	53.6 (8.9)	62.0 (8.4)	51.3 (7.6)

Data are presented as numbers or mean (SD).

**Table 2:** **Toe grip strength (TGS) and foot structure characteristics**

	**Total**	**Men**	**Women**	***p*** **value**
TGS (kg)	13.2 (5.6)	17.6 (6.0)	12.4 (5.3)	< 0.001
Foot length (cm)	22.7 (1.1)	24.6 (0.3)	22.3 (0.8)	< 0.001
Flexed foot length (cm)	19.8 (1.0)	21.5 (0.4)	19.5 (0.8)	< 0.001
Truncated foot length (cm)	17.2 (1.0)	18.4 (0.8)	17.0 (0.8)	< 0.001
Navicular height (cm)	4.8 (0.6)	5.1 (0.7)	4.7 (0.5)	< 0.001
HVA (°)	18.8 (7.5)	16.3 (2.3)	19.3 (8.0)	0.065
Toe curl ability (%)	12.5 (4.4)	12.6 (1.0)	12.5 (4.7)	0.683
FAH (%)	27.8 (3.3)	27.8 (4.2)	27.8 (3.2)	0.709

All data are expressed as mean (SD).

HVA, Hallux valgus angle; Toe curl ability (foot length – flexed foot length)/foot length; FAH, Foot arch height, Navicular height/base of arch.

According to Pearson’s correlation coefficient, TGS was significantly correlated with age (*p* < 0.01), height (*p* < 0.05), toe curl ability (*p* < 0.01), and FAH (*p* < 0.05), but not with weight or HVA, in men (Table 3). In women, TGS was significantly correlated with age (*p* < 0.01), height (*p* < 0.01), and toe curl ability (*p* < 0.01), but not with weight, HVA, or FAH (Table 3).

**Table 3:** **Correlation coefficients between toe grip strength and other factors, according to sex**

	**Age**	**Height**	**Weight**	**HVA**	**Toe curl ability**	**FAH**
Men	−0.43^**^	0.32^*^	0.26	0.07	0.39^**^	−0.33^*^
Women	−0.56^**^	0.40^**^	0.12	−0.08	0.44^**^	0.04

^*^p < 0.05, ^**^p < 0.01.

HVA, Hallux valgus angle; FAH, Foot arch height.

In the stepwise multivariate linear regression analyses, age, height, toe curl ability, and FAH were applied as the explanatory variables, for men. TGS was shown to be correlated with age (standardised partial regression coefficient [β]=−0.339, *p*–0.015) and toe curl ability (β–0.282, *p*–0.042), but not with height or FAH; the TGS adjusted R^2^ was 0.22 (Table 4). For women, age, height, and toe curl ability were applied as the explanatory variables. TGS was correlated with age (β–−0.397, *p* < 0.001), height (β–0.167, *p*–0.011), and toe curl ability (β–0.283, *p* < 0.001); the adjusted R^2^ was 0.40 (Table 5).

**Table 4:** **Stepwise multiple regression analysis of toe grip strength in men**

	**Regression coefficient**	**Standard error**	**β**	***P*** **value**
Intercept	21.585	4.559		< 0.001
Age (years)	−0.136	0.054	−0.339	0.015
Toe curl ability (%)	0.417	0.199	0.282	0.042

Explanatory variables were age, height, toe curl ability, and FAH.

β: standardised partial regression coefficient.

Adjusted R^2^=0.224.

**Table 5:** **Stepwise multiple regression analysis of toe grip strength in women**

	**Regression coefficient**	**Standard error**	**β**	**P value**
Intercept	−1.568	8.974		0.861
Age (years)	−0.155	0.026	−0.397	< 0.001
Height (cm)	0.138	0.054	0.167	0.011
Toe curl ability (%)	0.304	0.066	0.283	< 0.001

Explanatory variables were age, height, and toe curl ability.

β: standardised partial regression coefficient.

Adjusted R^2^=0.404.

## Discussion

This study investigated the relationships between TGS and HVA, toe curl ability, and FAH, and indicated that there is an association between TGS and toe curl ability in both men and women. Further, the results indicate that improving toe curl ability, i.e., the range of toe flexion motion and arch contraction, may improve TGS. Therefore, range-of-motion or joint mobilisation exercises that improve the range of toe flexion or toe curl ability, as well as muscle strengthening exercises, might improve TGS. However, at present, a causal relationship between TGS and toe curl ability cannot be demonstrated. Whether or not improving toe curl ability leads to improved TGS will have to be investigated in a future study.

Based on the Pearson’s correlation coefficients, TGS was negatively correlated with age and positively correlated with height and toe curl ability, in both sexes. The correlations between TGS and the demographic and clinical characteristics, for both sexes, were similar to the results observed in our previous study [6], except for weight. The correlation between TGS and toe curl ability suggests that the active range of toe and foot motion generated the TGS because toe curl ability positively correlated with TGS, in both sexes. In men, TGS negatively correlated with FAH, indicating that increased arch height resulted in decreased TGS. Moreover, in men, FAH was positively correlated with age and negatively correlated with height and toe curl ability (data not shown). Therefore, there might be other confounding factors affecting the relationship between TGS and FAH.

According to the stepwise multivariate linear regression analyses, TGS correlated with age and toe curl ability, in both sexes. In addition, height was shown to correlate with TGS in females. In our previous study, 31% of TGS was accounted for based on age, sex, height, and weight. In this study, the stepwise multivariate linear regression analyses were calculated based on age, height, and toe curl ability, in women, and on age, height, toe curl ability, and FAH, in men. As a result, 22% of the TGS was accounted for by age and toe curl ability, in men. In women, 41% of the TGS was accounted for by age, height, and toe curl ability. The reason for the differences in the adjusted R^2^ values for the stepwise multivariate linear regression analyses between the present and previous studies might be related to the inclusion of sex as one of the explanatory variables. In the future, factors other than foot characteristics will require more detailed investigation to clarify the factors contributing to TGS; factors such as exercise, sports history, and occupation may also influence TGS.

Age demonstrated the strongest correlation with TGS in both sexes. This result was consistent with that of our previous study [6], indicating that age-related changes are the strongest contributing factors of TGS, regardless of body type or foot characteristics.

TGS correlated with toe curl ability, but not with HVA or FAH. Therefore, toe curl ability is an important contributing factor for TGS. In the present study, toe curl ability (percentage) was calculated as (foot length–flexed foot length)/foot length. This calculation considers the sum of the range of motion of the first metatarsophalangeal and interphalangeal joints, as well as some degree of intertarsal mobility. When measuring TGS, the participants were asked to pull the grip bar using their toes; in other words, they were required to curl their toes around the grip bar. Theoretically, flexible toes should generate higher TGS. However, the toe curl ability values, in the present study, did not represent the ability of all the toes because the toe curl ability was calculated based on the first toe. Since the muscle strength of the first toe has been reported to show the strongest association with TGS [17], the first toe was used as a benchmark to calculate toe curl ability. Future studies will need to consider the function of the other toes, as well.

In the present study, HVA was not significantly associated with TGS, but the toe flexor strength of individuals with hallux valgus has been reported to be weaker than that in normal individuals [7]. In this study, we did not analyse the individuals with severe hallux valgus who could not correctly grip the toe grip dynamometer bar. Therefore, the HVA might not have influenced the TGS in the present study.

The FAH was negatively correlated with TGS in men, but was not correlated with TGS in women. We hypothesised that arch height might be positively correlated with TGS because the flexor hallucis longus and flexor digitorum longus muscles and other intrinsic foot muscles support the MLA. As previously mentioned, weak plantar intrinsic or extrinsic muscles (i.e., toe flexors) cannot provide sufficient dynamic truss support for the MLA [8]. In addition, the force produced by the intrinsic muscles was previously reported to contribute to the truss support of the MLA [8]. Headlee et al. [10] reported that plantar intrinsic foot muscle fatigue increases navicular drop. This report also indicated a relationship between toe flexor strength and the MLA. Jung et al. [18] showed that toe curl exercises do not change the MLA angle formed by the line connecting the first metatarsal head and the navicular tuberosity and the line connecting the navicular tuberosity and the medial side of the calcaneal bone. However, they also reported that the MLA angle significantly decreased during short-foot exercises, which are performed to activate the foot’s intrinsic muscles by pulling the metatarsal heads toward the heel, while the long toe flexors are relaxed. These results suggest that toe curls, such as during TGS measurements, may not influence arch height.

This study has several limitations. First, the participants were healthy and free of major foot problems. Furthermore, the participants were 20 to 79-years-old and all were Japanese. Therefore, our results cannot be generalised beyond this population. Western people tend to wear shoes indoors, whereas Japanese do not. This cultural difference may have an effect on foot structure and function, and on TGS. Second, the number of male participants was relatively small compared to the number females. Additionally, more than the half the participants were 60 to 79 years of age. Future studies will require a larger and more evenly age-distributed population. Finally, the TGS and characteristics were measured in a sitting position, which is a non-weight bearing position. We measured TGS in the sitting position using the toe grip dynamometer because standing TGS measurements are difficult, especially for older participants. Thus, we will also investigate TGS and foot characteristics in the standing position in a future study.

## Conclusion

This study investigated the relationships between TGS and HVA, toe curl ability, and FAH. TGS was associated with toe curl ability, but not with HVA or FAH, in both sexes. The results of this study suggest that improving the range of toe flexion and/or toe curl ability may strengthen TGS. However, to clarify the factors that contribute to TGS, factors other than foot characteristics will need to be investigated in a larger population.

## Abbreviations

TGS: Toe grip strength
MLA: Medial longitudinal arch
HVA: Hallux valgus angle
FAH: Foot arch height
